# Supplementary material for: Transient mTOR inhibition rescues 4-1BB CAR-Tregs from tonic signal-induced dysfunction
Source: Nat Commun. 2021 Nov 8;12:6446. doi: 10.1038/s41467-021-26844-1 (PMC8575891; doi:10.1038/s41467-021-26844-1)
Supplement: Supplementary file 1 — Supplementary Information [file 41467_2021_26844_MOESM1_ESM.pdf]

SUPPLEMENTARY INFORMATION

Supplementary Figures

- Supplementary Figure 1 ……………. p2
- Supplementary Figure 2 ……………. p3
- Supplementary Figure 3 ……………. p4
- Supplementary Figure 4 ……………. p5
- Supplementary Figure 5 ……………. p6
- Supplementary Figure 6 ……………. p7
- Supplementary Figure 7 ……………. p8
- Supplementary Figure 8 ……………. p9

Supplementary Tables

- Supplementary Table 1 ……………. p10
- Supplementary Table 2 ……………. p10

Supplementary Figure 1

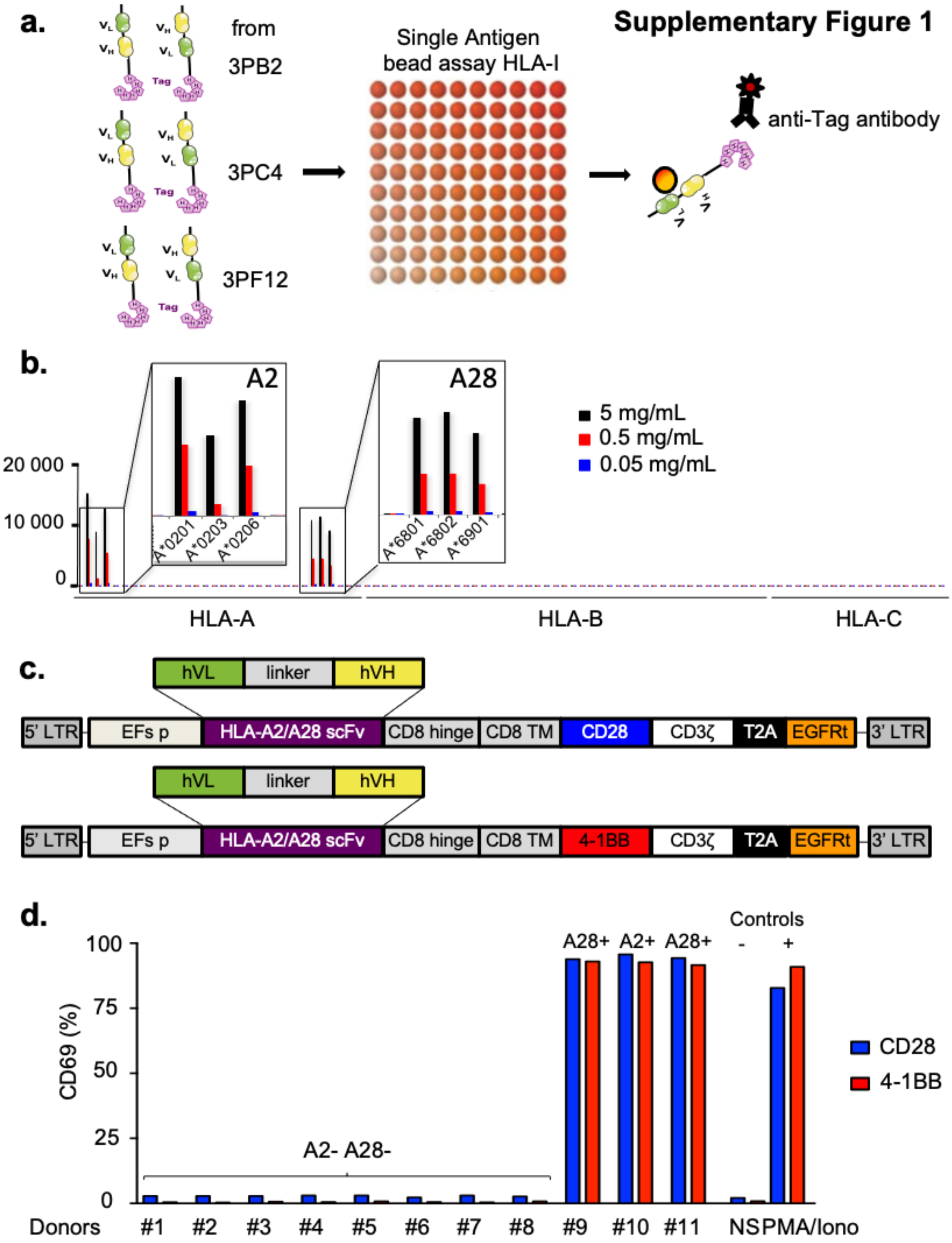

Supplementary Figure 1: Selection of specific scFvs for CD28 and 4-1BB CAR constructs.

a-b. Six tagged scFvs were tested against the Luminex single antigen bead assay (class I HLA) at different concentrations and specificity was assessed after PE-conjugated anti-tag staining. **a**. Schematic design of the experiment. **b**. Histogram representing the specificity of selected scFvs at different concentrations. **c**. CD28 and 4-1BB CAR constructs with the same framework and scFv against HLA-A2 and HLA-A28. **d**. Histogram representing the frequency of activated CAR-J.RT3-T3.5 cells (CD69-positive cells) after 24h of co-culture with splenocytes according to HLA type.

Supplementary Figure 2

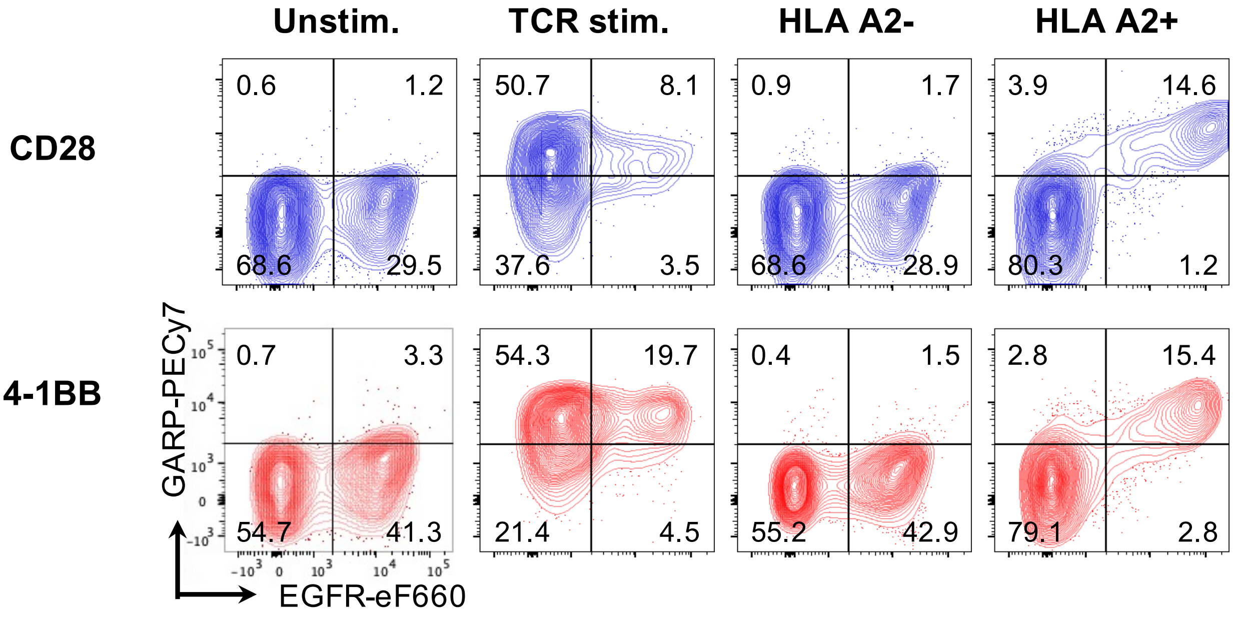

Supplementary Figure 2: CAR stimulation induces GARP expression.

On day 11, CD28 and 4-1BB CAR-Tregs were incubated with or without anti-CD3/CD28 beads (TCR stim.) or HLA-A2-positive or HLA-A2-negative irradiated splenocytes. GARP and EGFR expressions were measured by flow cytometry. Contour plots are representative of 2 experiments.

Supplementary Figure 3

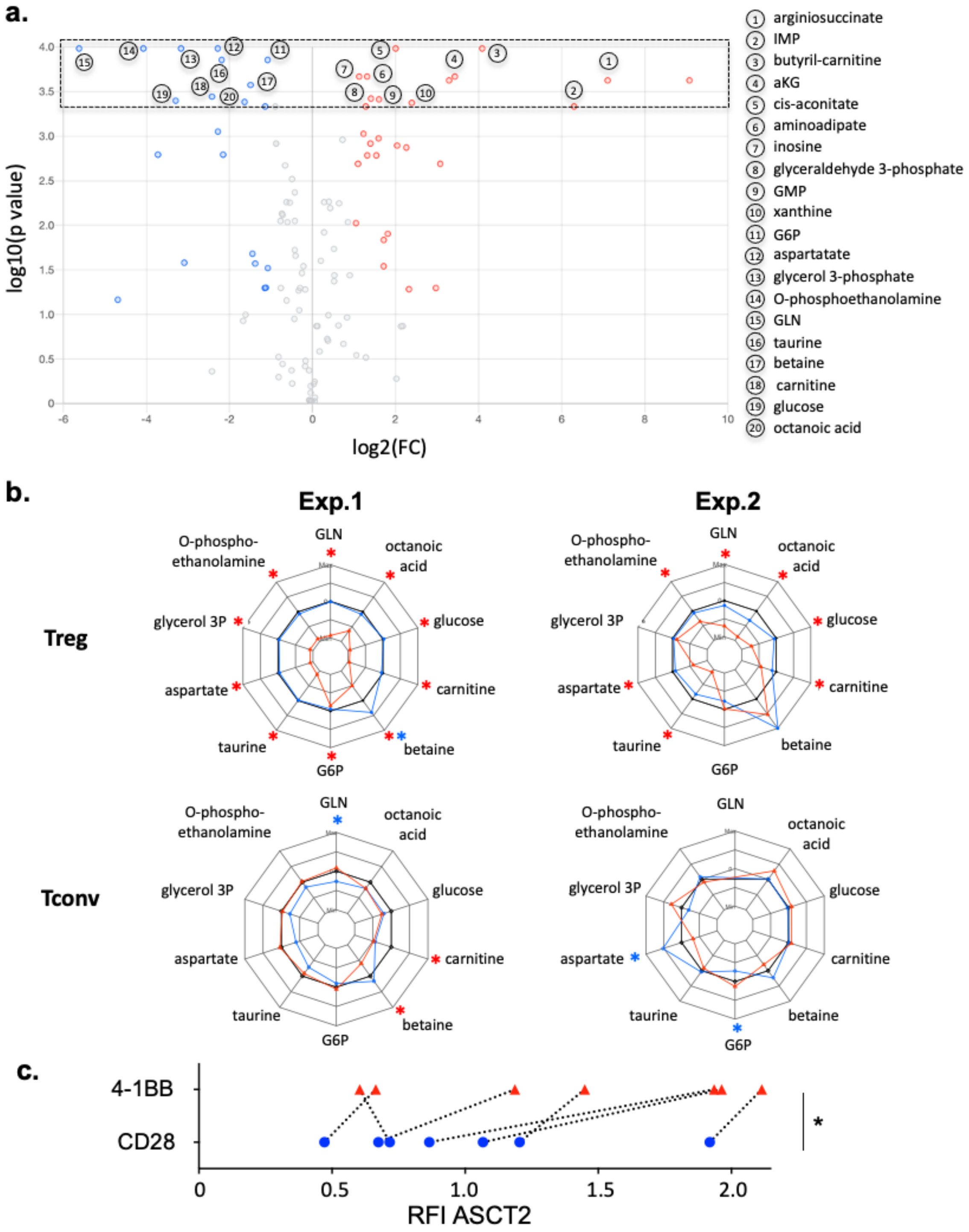

Supplementary Figure 3: 4-1BB tonic signal-induced metabolic changes.

a. Volcano plot representing the fold change of metabolites detected in untransduced (blue) versus 4-1BB CAR-Tregs (red). The top 20 differentially expressed metabolites are indicated. **b.** Radar plots representing the fold change of the indicated metabolites in CD28 (blue), 4-1BB (red) and untransduced (black) CAR-Tregs (upper panels) or CAR-Tconvs (lower panels) of two independent experiments. **c.** On day 16, ASCT2 expression was measured by flow cytometry. The ratios of CD28 CAR-Tregs/UT and 4-1BB CAR-Tregs/UT of 7 independent experiments are shown. Two-tailed Wilcoxon matched-pairs signed rank test *p<0.05. Exact p-value = 0.0312.

Supplementary Figure 4

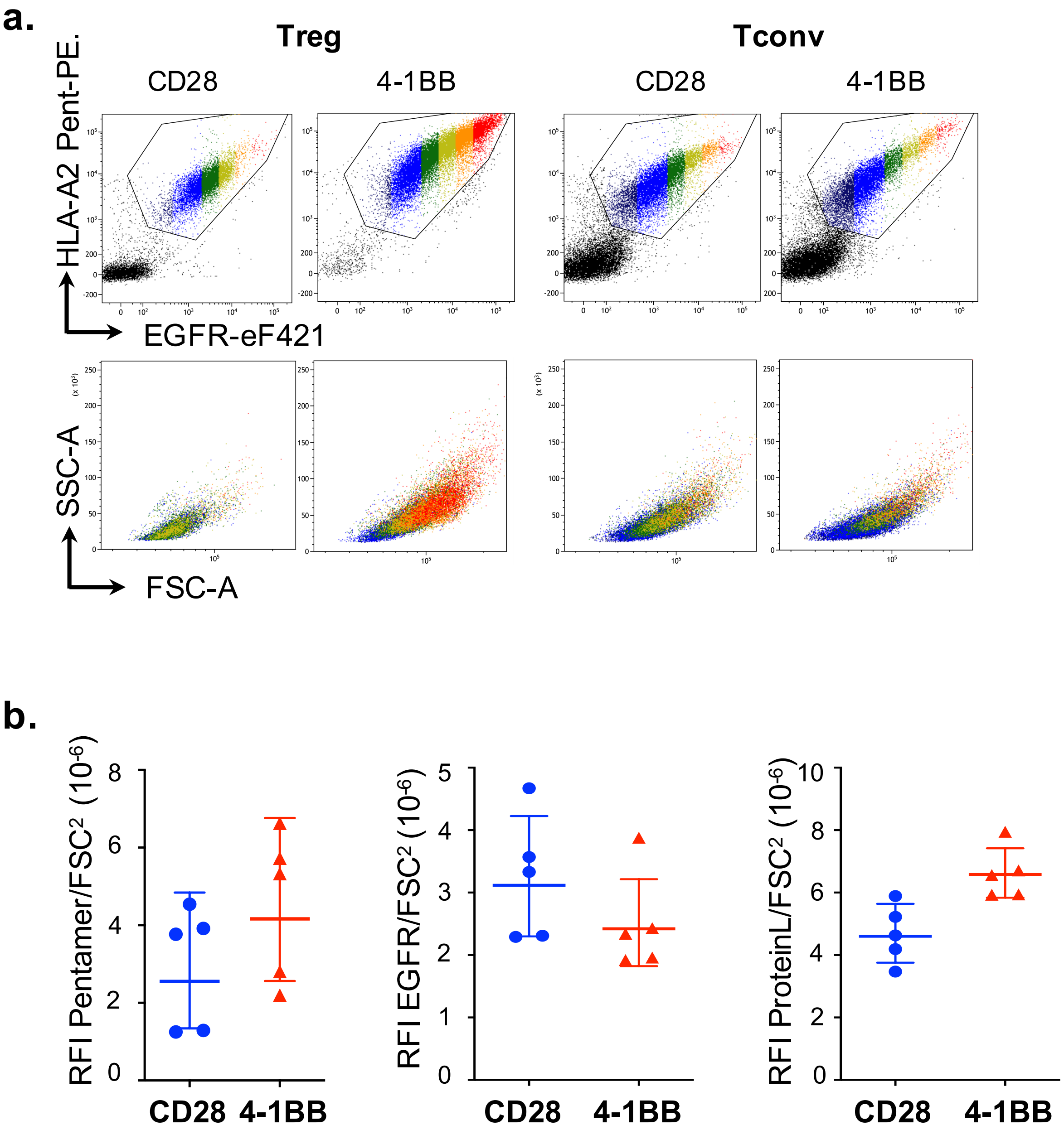

Supplementary Figure 4: Correlation between blastic phenotype and increased CAR/EGFRt expression.

a. Flow cytometry analysis of HLA-A2 pentamer binding and EGFRt reporter gene expression (upper panel) and side and forward (lower panel) scatter parameter MFIs of 4-1BB and CD28 CAR-Tregs on day 11 of culture. A color scale was applied to gate transduced cells. Dot plots are representative of 7 experiments. **b**. Ratios of pentamer MFI (left panel), EGFR MFI (middle panel) and protein L MFI (right panel) normalized to CAR-Treg cell surface estimated by as squared FSC are represented and correspond to 5 independent experiments. Geometric means with geometric SD are shown.

Supplementary Figure 5

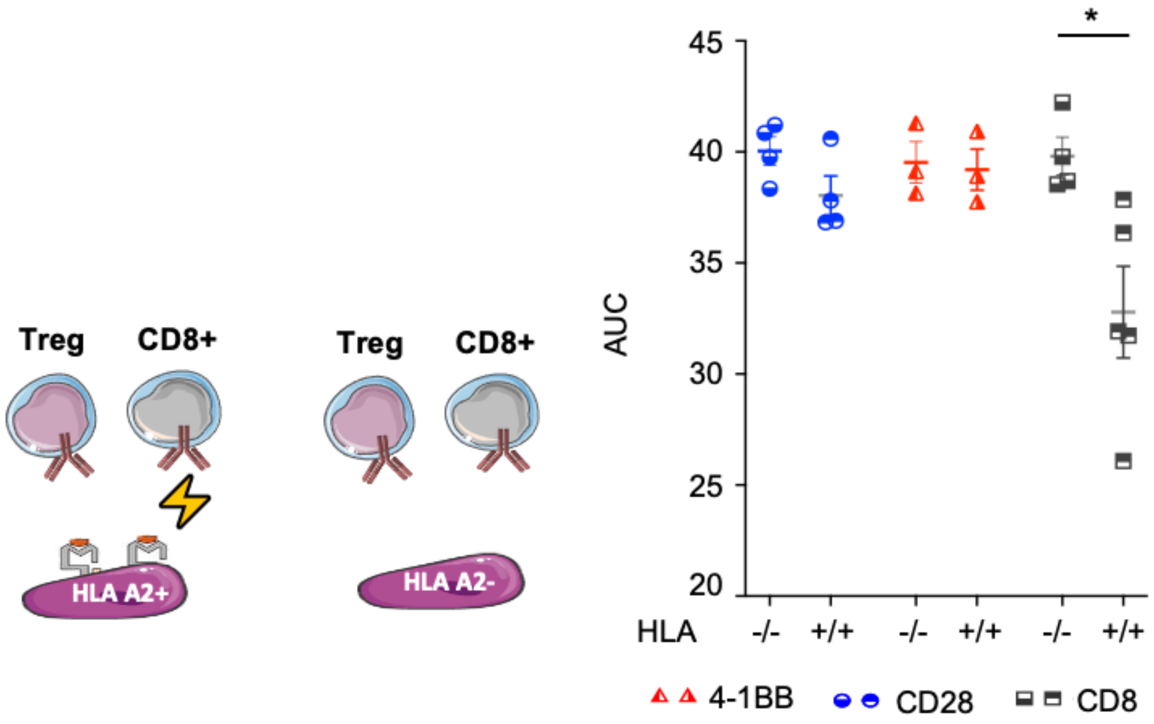

Supplementary Figure 5: 4-1BB CSD does not induce cytotoxic CAR Tregs.

Scheme of the xCELLigence cytotoxicity assay. HLA-A2+ endothelial cells (ECs) or HLA-A2- ECs were cocultured with CD28 (n=4) or 4-1BB CAR-Treg (n=3) or cytotoxic CD8+ CAR-Tconv cells (n=5 with HLA-A2+; n=4 with HLA-A2-). The areas under the curve (AUCs) from 4 independent experiments are shown. Mean +/- SEM is depicted. Two-tailed Mann-Whitney test. *p<0.05. Exact p-value = 0.0159

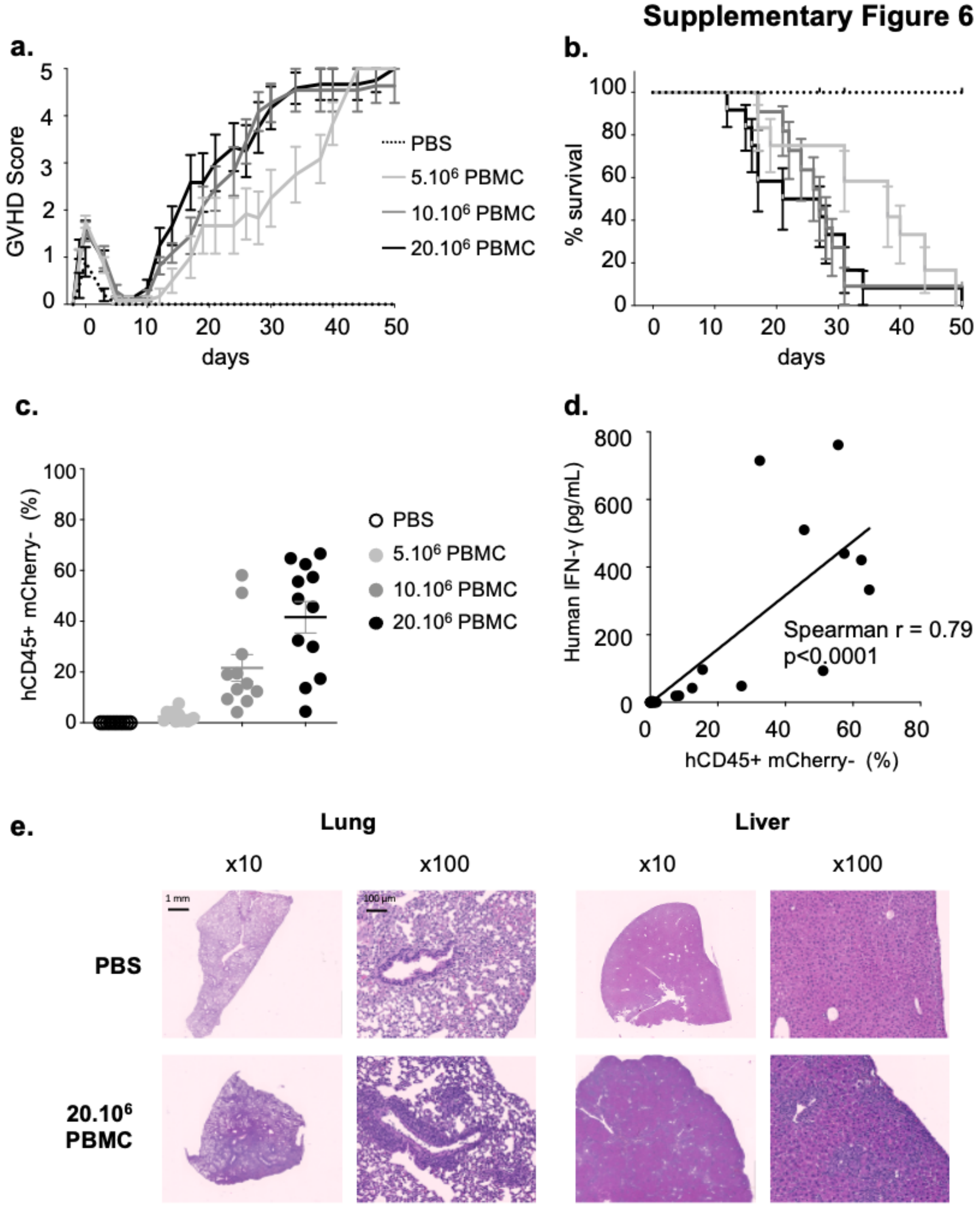

Supplementary Figure 6: XenoGVHD model using the busulfan conditioning regimen.

Eight- to 12-week-old male NSG mice were conditioned with busulfan on day -2 and day -1 before IV injection with the indicated number of HLA-A2+ PBMCs (PBS n=10, 5.10^{6} PBMC n=12, 10.10^{6} PBMC n=11 and 20.10^{6} PBMC n=12). Mice were weighed and scored for GVHD 3 times weekly and bled weekly for flow cytometry analysis. **a**. GVHD score. **b**. Survival curves. **c**. Ten days post cell injection, the proportion of hCD45+ cells among total CD45+ cells (hCD45+ and mCD45+) was determined by flow cytometry. **d**. Ten, 17 and 24 days post-cell injection, the concentration of human IFNγ in the plasma was measured using cytometry bead array and correlated with the human chimerism. **e**. Representative anatomopathological analysis of lesions in the liver and lung performed at day 30 in NSG mice injected with 20 x 10^{6} HLA-A2+ PBMCs. Mean+/-SD is represented. N=3 mice per group from at least 3 independent experiments and 3 different donors.

Supplementary Figure 7

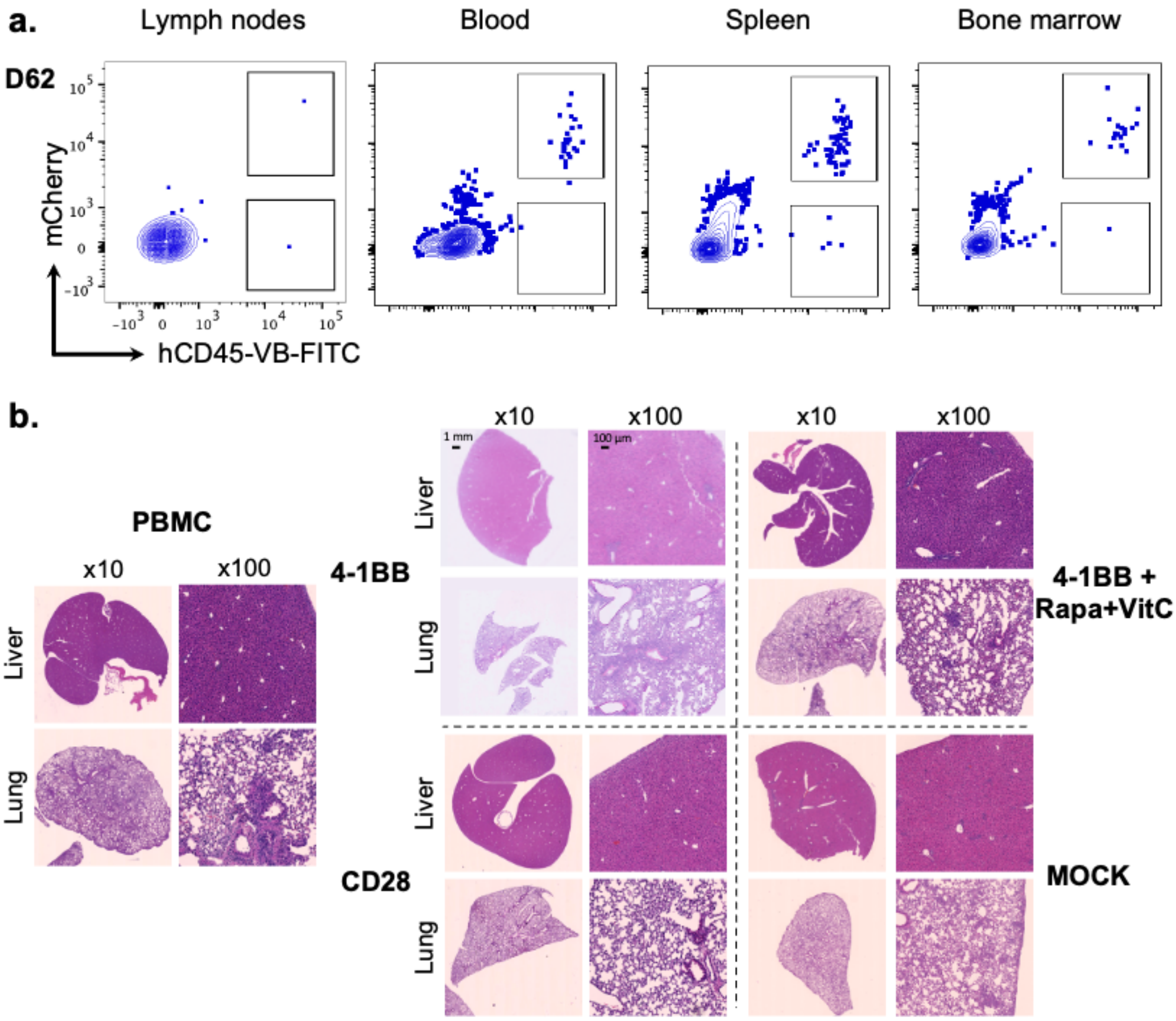

Supplementary Figure 7: CAR-Tregs detection in the xenoGVHD model.

a. Representative contour plots at sacrifice (day 60-62) of murine and human cells in the blood, spleen and bone marrow. **b**. Representative pathological features of infiltrates in liver and lung at sacrifice (either when mouse reached GVHD score>4 or at day 60 corresponding to the end of experiment) in NSG mice injected with 5 x10^6 HLA-A2+ PBMC followed by retro orbital injection of 5 x10^6 of the indicated type of Tregs.

Supplementary Figure 8

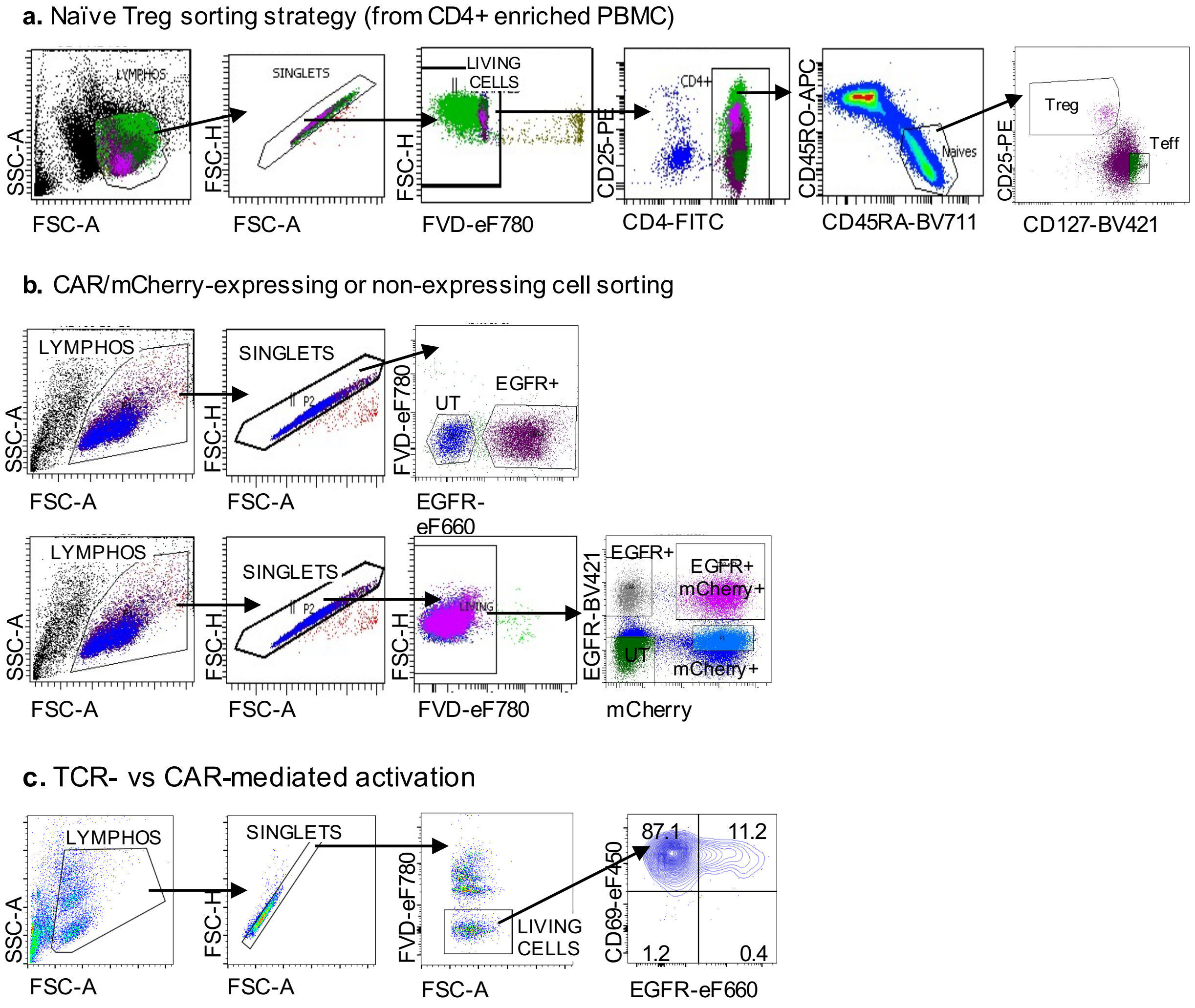

Supplementary Figure 8. **Gating strategies used for cell sorting and FACS analyses.**
a. Gating strategy to sort Treg (CD4+CD45RO-CD45RA+CD25+CD127-) and Tconv (CD4+CD45RO-CD45RA+CD25-CD127+) cells from CD4-positive-enriched PBMCs, depicted on Figure 1b. **b.** Gating strategy to sort CAR-expressing (EGFR+) or non-expressing (EGFR-) cells, or CAR/Luciferase-expressing (EGFR+mCherry+) or single transduced (EGFR+ or mCherry+) cells referred to as CAR-Treg sorting in Figure 1c. **c.** Gating strategy for FACS analysis of TCR/CAR-mediated CAR-Treg activation depicted on Figure 1d.
The same strategy was used at D16 of culture for tSNE mapping of CD28 and 4-1BB CAR-Tregs (Figure 3a), CD28 and 4-1BB CAR-Treg or CAR-Tconv stability (Figure 4a), phosphoS6 staining in 4-1BB CAR-Tregs cultured with or without rapamycin and vitamin C (Figure 5a), TCR/CAR-mediated activation assay in UT or CAR-Tregs (Figure 8a, 8b).
Abbreviation: LYMPHOS = Lymphocytes

Supplementary Table 1: qPCR and digital droplet PCR primers

	Primer denomination	target	5' > 3' primer sequence
CAR transcripts by qPCR	qPCR_scFv_10F	scFv	TCAGCAGAAGCCCGGCAAG
	qPCR_scFv_10R		TCCGCCAAAGGTCAGTGGGA
	qPCR_EGFRt_9F	EGFRt reporter gene	GACTGCGTCTCTTGCCGGAAT
	qPCR_EGFRt_9R		AAGGTTGCACTTGTCCACGCA
	HPRT F	HPRT housekeeping gene	TTGCTTTCCTTGGTCAGGCA
	HPRT R		ATCCAACACTTCGTGGGGTC
VCN calculation by ddPCR	hAlb-F	Albumin housekeeping gene	ACTCATGGGAGCTGCTGGTTC
	hAlb-R		GCTGTCATCTCTTGTGGGCTGT
	hAlb-Probe (VIC)		CCTGTCATGCCCACACAAATCTCTCC
	Psi-F	viral Psi sequence (encapsidation sequence)	CAGGACTCGGCTTGCTGAAG
	Psi-R		TCCCCCGCTTAATACTGACG
	Psi-Probe (FAM)		CGCACGGCAAGAGGCGAGG

Supplementary Table 2: List of antibodies and staining reagents

Name	Fluorophore	Clone	Manufacturer	Catalog number	Dilution
anti-Mouse IgG (H+L)	Alexa Fluor 647		ThermoFisher Scientific	A31571	1/100
ASCT2 Fc fusion RBD	unconjugated	SLC1A5	Metafora	ASCT2-M25	1/50
CCR7	PE Cy7	3D12 (RUO)	BD Biosciences	557648	1/20
CD107a	Brillant Violet 786	H4A3	BD Biosciences	563869	1/20
CD127	Brillant Violet 421	HIL-7R-M21	BD Biosciences	562436	1/50
CD137	Brillant Violet 605	4B4-1	Sony biotechnology	2149140	1/20
CD152	PE Cy5	BNI3	BD Biosciences	555854	1/5
CD15s	Brillant Violet 510	CSLEX1	BD Biosciences	563529	1/20
CD25	PE	M-A251	BD Biosciences	555432	1/10
CD278	PerCPCyanine5.5	C398.4A	Ozyme	313518	1/25
CD4	FITC	SK3/SK4	BD Biosciences	347413	1/5
CD45RA	Brillant Violet 711	HI100	Ozyme	304138	1/50
CD45RO	APC	UCHL1	BD Biosciences	559865	1/25
CD69	eFluor 450	FN50	ThermoFisher Scientific	48-0699-42	1/25
CD8	PerCP Cy5,5	RPA-T8	BD Biosciences	560662	1/20
EGFR	eFluor 660	ME1B3	ThermoFisher Scientific	50-9509-42	1/25
FOXP3	PE	PCH101	ThermoFisher Scientific	12-4776-42	1/20
FVD eF780	eFluor 780		ThermoFisher Scientific	65-0865-18	1/1000
Granzyme B	Brillant Violet 510	GB11	BD Biosciences	563388	1/20
CD45	VioBright FITC	5B1	Miltenyi Biotec	130-104-517	1/50
HELIOS	eFluor 450	21F6	ThermoFisher Scientific	48-9883-42	1/25
HLA-A2/A28	FITC		OneLambda	FH0037	1/10
HLA-DR	APC-R700	G46-6	BD Biosciences	565127	1/20
Ki67	FITC	B56 and MIB 1	BD Biosciences	556028	1/5
mouse CD45	V450	30-F11	BD Biosciences	560501	1/33
Phospho-S6 Ribosomal Protein (Ser235/236)	Alexa Fluor 700	D57.2.2E	Ozyme	27036S	1/50
Pro5 MHC Pentamer A2	PE		ProImmune	F010-2A-E	1/20
Protein L	biotinylated		Thermo Scientific	29997	1/1500
Streptavidin	Brillant Violet 421		BD Biosciences	563259	1/1000
TIGIT	APC	A15153G	Sony biotechnology	2463530	1/20
