# Supplementary material for: Transient mTOR inhibition rescues 4-1BB CAR-Tregs from tonic signal-induced dysfunction
Source: Nat Commun. 2021 Nov 8;12:6446. doi: 10.1038/s41467-021-26844-1 (PMC8575891; doi:10.1038/s41467-021-26844-1)
Supplement: Supplementary file 3 — Reporting summary [file 41467_2021_26844_MOESM3_ESM.pdf]

nature portfolio

Corresponding author(s): ZUBER julien

Last updated by author(s): 2021/09/14

Reporting Summary

Nature Portfolio wishes to improve the reproducibility of the work that we publish. This form provides structure for consistency and transparency in reporting. For further information on Nature Portfolio policies, see our Editorial Policies and the Editorial Policy Checklist.

Statistics

For all statistical analyses, confirm that the following items are present in the figure legend, table legend, main text, or Methods section.

n/a	Confirmed	
☐	☒	The exact sample size (*n*) for each experimental group/condition, given as a discrete number and unit of measurement
☐	☒	A statement on whether measurements were taken from distinct samples or whether the same sample was measured repeatedly
☐	☒	The statistical test(s) used AND whether they are one- or two-sided<br>*Only common tests should be described solely by name; describe more complex techniques in the Methods section.*
☒	☐	A description of all covariates tested
☐	☒	A description of any assumptions or corrections, such as tests of normality and adjustment for multiple comparisons
☐	☒	A full description of the statistical parameters including central tendency (e.g. means) or other basic estimates (e.g. regression coefficient) AND variation (e.g. standard deviation) or associated estimates of uncertainty (e.g. confidence intervals)
☐	☒	For null hypothesis testing, the test statistic (e.g. *F*, *t*, *r*) with confidence intervals, effect sizes, degrees of freedom and *P* value noted<br>*Give P values as exact values whenever suitable.*
☒	☐	For Bayesian analysis, information on the choice of priors and Markov chain Monte Carlo settings
☒	☐	For hierarchical and complex designs, identification of the appropriate level for tests and full reporting of outcomes
☒	☐	Estimates of effect sizes (e.g. Cohen's *d*, Pearson's *r*), indicating how they were calculated

Our web collection on statistics for biologists contains articles on many of the points above.

Software and code

Policy information about availability of computer code

Data collection	Data were collected using GraphPad Prism software (version 8.00; GraphPad Software)
Data analysis	Analyses were performed using GraphPad Prism software (version 8.00; GraphPad Software). For statistical comparisons of in vitro data, we used the nonparametric two-tailed Mann-Whitney test for comparisons of two groups. For survival comparisons, the log-rank test was used. P-values <0.05 were considered significant. Autoscaled metabolic data were normalized by median through Metaboanalyst 5.0 and compared with FDR correction. For radar plots and volcano plots, RStudio (version 3.6.3) and the following R packages were used: dplyr, ggrepel, ggplot2, fmsb and scales packages.

For manuscripts utilizing custom algorithms or software that are central to the research but not yet described in published literature, software must be made available to editors and reviewers. We strongly encourage code deposition in a community repository (e.g. GitHub). See the Nature Portfolio guidelines for submitting code & software for further information.

Data

Policy information about availability of data

All manuscripts must include a data availability statement. This statement should provide the following information, where applicable:

- Accession codes, unique identifiers, or web links for publicly available datasets
- A description of any restrictions on data availability
- For clinical datasets or third party data, please ensure that the statement adheres to our policy

ATA AVAILABILITY (p. 39)
The authors declare that all data supporting the findings of this study are available in the article and its Supplementary Information Files, or upon request from the corresponding author. The data underlying all the figures and Supplementary Table 1 and Supplementary Table 2 are provided as a source data file. The

transcriptomic data reported in this study have been deposited in the Gene Expression Omnibus (GEO) database under accession no. GSE183598 (human RNAseq) and can be accessed through the hyperlink: https://www.ncbi.nlm.nih.gov/geo/info/linking.html. Metabolomics data have been deposited to the EMBL-EBI MetaboLights database (DOI: 10.1093/nar/gkz1019, PMID:31691833) with the identifier MTBLS3121.
The complete dataset can be accessed here https://www.ebi.ac.uk/metabolights/MTBLS3121.

Field-specific reporting

Please select the one below that is the best fit for your research. If you are not sure, read the appropriate sections before making your selection.

- [x] Life sciences
- [ ] Behavioural & social sciences
- [ ] Ecological, evolutionary & environmental sciences

For a reference copy of the document with all sections, see nature.com/documents/nr-reporting-summary-flat.pdf

Life sciences study design

All studies must disclose on these points even when the disclosure is negative.

Sample size	For this project, we developed a new GVHD mouse model and we could hardly predict the position (mean) and dispersion (SD) parameters for human chimerism and survival. Similarly, we had no hint about the efficacy of our CAR-Tregs nor had we about expected differences between the two CAR constructs. Hence, we did not make any calculation to estimate animal numbers. Replication of experiments allowed us to include at least 10 animals per experimental group.
Data exclusions	Three Treg cultures were interrupted and excluded from further analysis because the FOXP3+ frequency had dropped below 50% in the untransduced Treg population at early time point (Day 10). Regardless the cause (IL-2, donor, sorting quality), we considered that these experiments would not allow comparison between CAR-Tregs and untransduced (control) Tregs. This point is indicated in the revised M&M (p. 28).
Replication	All results presented here have been replicated and robustly confirmed in separate experiments. However, as indicated in the manuscript, the metabolic and transcriptomic analyses include only two replicates, which are very consistent with each other. To further stress this point, the two independent metabolic experiments are disclosed separately.
Randomization	All animals were male to control for this important covariate. Moreover, the animals were randomly picked in each cage and allocated to a treatment group in order to control for litter and age heterogeneity across the experimental groups.
Blinding	The histological analysis (GVHD scoring) was performed in a blind manner by two separate experimenters.

Reporting for specific materials, systems and methods

We require information from authors about some types of materials, experimental systems and methods used in many studies. Here, indicate whether each material, system or method listed is relevant to your study. If you are not sure if a list item applies to your research, read the appropriate section before selecting a response.

Materials & experimental systems

n/a	Involved in the study	
[ ]	[x]	Antibodies
[ ]	[x]	Eukaryotic cell lines
[x]	[ ]	Palaeontology and archaeology
[ ]	[x]	Animals and other organisms
[x]	[ ]	Human research participants
[x]	[ ]	Clinical data
[x]	[ ]	Dual use research of concern

Methods

n/a	Involved in the study	
[x]	[ ]	ChIP-seq
[ ]	[x]	Flow cytometry
[x]	[ ]	MRI-based neuroimaging

Antibodies

Antibodies used	The list of antibodies is provided as Supplementary Information
Validation	All the antibodies used in this study were titrated and tested on positive and negative cells upon reception from the manufacturer.

Eukaryotic cell lines

Policy information about cell lines

Cell line source(s)	The source of the CiGenc cell line is indicated p. 37

Authentication	The HLA +/+ and HLA -/- CiGEnc cell lines were tested for HLA A2 expression before use.
Mycoplasma contamination	All cell lines tested negative for Mycoplasma contamination. Mycoplasma testing is performed routinely every 3 months for all cell lines at our lab.
Commonly misidentified lines (See ICLAC register)	*Name any commonly misidentified cell lines used in the study and provide a rationale for their use.*

Animals and other organisms

Policy information about studies involving animals; ARRIVE guidelines recommended for reporting animal research

Laboratory animals	The strain, age and sexe of the animals are indicated in the M&M section (p. 36)
Wild animals	This study did not involve wild animals
Field-collected samples	This study did not involved field-collected samples.
Ethics oversight	Animal procedures were approved by the “Services Vétérinaires de la Préfecture de Police de Paris” and by the “Comité d'Ethique en matière d'Expérimentation Animale Paris Descartes (CEEA 34)” under the number APAFIS#23742-2017091815321774 v9, Université Paris Descartes, Paris, France.

Note that full information on the approval of the study protocol must also be provided in the manuscript.

Flow Cytometry

Plots

Confirm that:

- [x] The axis labels state the marker and fluorochrome used (e.g. CD4-FITC).
- [x] The axis scales are clearly visible. Include numbers along axes only for bottom left plot of group (a 'group' is an analysis of identical markers).
- [x] All plots are contour plots with outliers or pseudocolor plots.
- [x] A numerical value for number of cells or percentage (with statistics) is provided.

Methodology

Sample preparation	Cell source and Treg purification process is detailed in the M&M section (p.27)
Instrument	Instruments used for FACS sorting and flow cytometry analysis are indicated in the manuscript (p.27)
Software	The softwares used for Flow Cytometry analysis are indicated in the Methods (p.29)
Cell population abundance	The sorting purity was always checked after FACS sorting and greater than 98% (as indicated p. 28).
Gating strategy	The gating strategies are shown in Figure 1b and supplementary figure 8

- [x] Tick this box to confirm that a figure exemplifying the gating strategy is provided in the Supplementary Information.
